# Tetrapeptides Modelled to the Androgen Binding Site of ZIP9 Stimulate Expression of Tight Junction Proteins and Tight Junction Formation in Sertoli Cells

**DOI:** 10.3390/biology11010055

**Published:** 2021-12-31

**Authors:** Marie-Louise Möller, Ahmed Bulldan, Georgios Scheiner-Bobis

**Affiliations:** Institute of Veterinary Physiology and Biochemistry, Justus Liebig University Giessen, Frankfurter Str. 100, 35392 Giessen, Germany; marie-louise.moeller@gmx.net (M.-L.M.); ahmedbulldan@yahoo.com (A.B.)

**Keywords:** ZIP9, androgen receptor, Sertoli cells, blood–testis barrier, tight junctions

## Abstract

**Simple Summary:**

Androgens such as testosterone act either through the nuclear androgen receptor or through the plasma membrane-bound androgen receptor ZIP9. Androgens are crucial for male fertility. In the testis they stimulate the formation of tight junctions between Sertoli cells, thereby creating an immune-privileged environment for the development of fertile sperm known as the blood–testis barrier. Androgens are also absolutely required for spermatogenesis. Nevertheless, when low testosterone is supplemented by exogenous testosterone (e.g., in testosterone replacement therapy), it triggers a loss of sperm, and thus and infertility, by a negative feed-back mechanism based on the hypothalamic-pituitary-gonadal axis. In order to investigate this discrepancy, the effects of small peptides that fit within the androgen binding site of ZIP9 were tested for their effects on the expression of proteins involved in the formation of tight junctions. Our results provide evidence that all three peptides tested act through ZIP9 as androgens and promote tight junction protein expression and tight junction formation. They can therefore act as surrogates of testosterone for the formation and maintenance of the blood–testis barrier.

**Abstract:**

Androgens stimulate the expression of tight junction (TJ) proteins and the formation of the blood–testis barrier (BTB). Interactions of testosterone with the zinc transporter ZIP9 stimulate the expression of TJ-forming proteins and promote TJ formation in Sertoli cells. In order to investigate androgenic effects mediated by ZIP9 but not by the nuclear androgen receptor (AR), the effects of three tetrapeptides fitting the androgen binding site of ZIP9 were compared with those induced by testosterone in a Sertoli cell line expressing ZIP9 but not the AR. Three tetrapeptides and testosterone displaced testosterone-BSA-FITC from the surface of 93RS2 cells and stimulated the non-classical testosterone signaling pathway that includes the activation of Erk1/2 kinases and transcription factors CREB and ATF-1. The expression of the TJ-associated proteins ZO-1 and claudin-5 was triggered as was the re-distribution of claudin-1 from the cytosol to the membrane and nucleus. Furthermore, TJ formation was stimulated, indicated by increased transepithelial electrical resistance. Silencing ZIP9 expression by siRNA prevented all of these responses. These results are consistent with an alternative pathway for testosterone action at the BTB that does not involve the nuclear AR and highlight the significant role of ZIP9 as a cell-surface androgen receptor that stimulates TJ formation.

## 1. Introduction

Actions of testosterone are mediated by at least two different pathways: In the classical pathway testosterone or its derivative dihydroxy-testosterone (DHT) bind in the cell cytosol to the soluble androgen receptor (AR), which, by being a ligand-activated transcription factor, dimerizes and translocates into the nucleus to control expression of various genes [1,2]. The second pathway originates at the plasma membrane and is propagated through signaling cascades that are also known to be activated by growth factors. The idea that the classical, cytosolic AR associates with the plasma membrane to mediate these events has been seriously challenged in recent years through comprehensive work showing that the androgen receptor responsible for the mediation of fast responses to testosterone is not the nuclear AR but the membrane-embedded protein ZIP9 [3,4].

ZIP9, a rather unusual protein, is both a Zn^2+^ transporter from the family of the ZRT, IRT-like proteins (ZRT = zinc-regulated transporter; IRT = iron-regulated transporter) and a testosterone receptor and signal transducer that propagates its cytosolic effects by interacting with G-proteins [5,6,7,8,9]. ZIP9-mediated testosterone effects are of physiological, pathophysiological, and possibly of clinical relevance and are entirely independent of testosterone effects mediated through the classical nuclear AR [5,6,7,8].

Testosterone, although it is a pleiotropic hormone with many actions in both sexes, is mostly associated with the establishment and maintenance of masculinity. This viewpoint is entirely justified, considering its undisputed importance for the establishment of male phenotype, behavior, and fertility. In male gonads, testosterone produced the Leydig cells is the main regulator of the integrity of the blood–testis barrier (BTB) [10,11,12]. The primary function of the BTB is to restrict the paracellular flow of water and nutrients across the Sertoli cell epithelia and at the same time to protect the developing haploid forms of male germ cells by establishing an immune-privileged environment [13,14].

Although the protection and maintenance of the BTB are essential for male fertility, and defects of the BTB lead to infertility, testosterone replacement therapy (TRP), which is applied when endogenous testosterone levels are low, is not necessarily a first-choice treatment. Along with a variety of side effects, including severe blood- and heart-associated conditions such as polycythemia or cardiac hypertrophy and myocardial infarction [15,16], exogenously applied testosterone also unfavorably affects male fertility. By inducing negative feedback on the hypothalamic–pituitary–gonadal axis that regulates the biosynthesis and release of gonadotropin-releasing hormone from the hypothalamus as well the biosynthesis and release of follicle-stimulating hormone and luteinizing hormone from the pituitary gland [17], exogenously applied testosterone impairs testicular androgen biosynthesis and germ cell production and therefore acts as a male contraceptive [18]. Nevertheless, despite its negative effect on spermatogenesis, exogenous testosterone positively affects the formation and maintenance of the BTB by stimulating the expression of tight junction (TJ) proteins [19]. Thus, it would be advantageous to segregate the positive from the negative effects that testosterone can have on male fertility.

In Sertoli cells, testosterone acts through ZIP9 to stimulate the non-classical testosterone signaling cascade, which, through the activation of Erk1/2, ATF-1, or CREB, leads to increased expression of claudin-1 and claudin-5 and to enhanced TJ formation [6]. Stimulation of this particular androgen receptor with suitable, ZIP9-specific ligands could potentially avoid any unwanted effects that are generated through the interactions of testosterone with the classical AR.

The androgen binding site of ZIP9 is localized on the extracellular surface of the membrane-bound protein [7]. By applying in silico calculations described in detail in a parallel publication [20], we designed tetrapeptides that bind specifically within the androgen binding site of ZIP9 and stimulate pro-androgenic effects, including mineralization in osteoblasts and myotube formation of myogenic cells [20]. The present study was designed to address the suitability and potential of these peptides to act on Sertoli cells as ZIP9-directed testosterone surrogates by triggering signaling events that stimulate the expression of TJ proteins and TJ formation.

## 2. Materials and Methods

### 2.1. Cell Culture of Rat-Derived Sertoli Cells

The rat-derived prepubertal Sertoli cell line 93RS2 [21] was chosen for the experiments because these cells express ZIP9 but not the classical AR. The 28th passage of the cells was used for the experiments. Cells were regularly checked for SOX9 expression, a Sertoli-specific marker [22]. Cells were cultured in a humidified incubator (37 °C, 5% CO_2_) in 1:1 DMEM:Ham’s F-12 medium with L-glutamine supplemented with 10% (*v*/*v*) fetal bovine serum (FBS) and 1% (*v*/*v*) penicillin/streptomycin. The medium was replaced every two days.

### 2.2. Peptides Used in the Study

The in silico methods that led to the identification of tetrapeptides that target the androgen binding site of ZIP9 are described in detail in a previous publication [20]. The three tetrapeptides used in this study were IAPG (Pep-1), GVSG (Pep-2), and GVVG (Pep-3). Synthesis and analysis of the peptides were carried out as described [20].

### 2.3. Preparation of Cell Lysates from 93RS2 Cells

A total of 1 × 10^5^ 93RS2 cells were grown in 10-cm culture dishes as described above. Cells were then incubated for 24 h with 1% FBS before treatment with various concentrations of the peptides dissolved in medium or testosterone dissolved in ethanol; equal amounts of ethanol were also added to the peptide-treated cells and to control incubations. After 30 min of incubation the medium was removed by aspiration, and cells were washed twice with ice-cold PBS (without Ca^2+^ or Mg^2+^; Thermo Fisher Scientific, Darmstadt, Germany) and then lysed in 400 µL of a commercially available cell lysis buffer (Cell Signaling Technology, Frankfurt, Germany) according to the protocol of the provider. Immediately before use, 1 μM PMSF, 1× protease inhibitor cocktail (Roche, Mannheim, Germany), and 2 µg/mL pepstatin were added to the lysis buffer. All lysis steps were carried out on ice. After 5 min of incubation, cells were detached from the dish with a cell scraper and the suspensions were transferred into reaction vials and sonicated 5 times for 1 sec each with intervals of 1 s. After centrifugation of the lysates at 4 °C for 10 min at 13,000× *g*, the protein concentration in the supernatants was determined using a bicinchoninic acid (BCA) protein assay reagent kit (Pierce, Rockford, IL, USA) with inclusion of the lysis buffer in the standard curve.

### 2.4. Western Blotting

For Western blotting, 10 µg of protein from cell lysates was separated by SDS-PAGE on slab gels containing 10% acrylamide and 0.3% *N*,*N*′-methylene-bis-acrylamide. Biotinylated proteins (Cell Signaling Technology) served as molecular weight markers. After electrophoresis proteins were electro-blotted onto PVDF membranes (Merck Chemicals GmbH, Schwalbach, Germany) for 30 min at 0.5 V/cm^2^. Protein bands corresponding to phospho-Erk1/2 were visualized by incubating the membranes with the appropriate primary antibody following the recommendations of the provider (Cell Signaling Technology) and subsequently with the appropriate secondary antibody of an enhanced chemiluminescence solution (consisting of p-coumaric acid, luminol, and H_2_O_2_ mixed with the buffer [23]). Horseradish peroxidase-conjugated anti-biotin IgG (Cell Signaling Technology) was included in the mixture containing the secondary antibody at a dilution of 1:2000 in order to detect the biotinylated molecular weight markers. The resulting chemiluminescence was recorded by exposure to film, which was analyzed with the software program ImageJ (freely available at http://rsbweb.nih.gov/ij/ (accessed on 31 December 2021)). Loading differences in the various Western blots were corrected by taking into consideration the optical density of bands corresponding to unphosphorylated Erk1/2 bands or total actin detected in Western blots that were run in parallel. All antibodies used in Western blots and their concentrations (dilutions) are summarized in Supplementary Table S1.

### 2.5. Immunofluorescence

Each chamber of 4-chamber slides was plated with 1 × 10^4^ 93RS2 cells and cultured as described above to approximately 80% confluence. Incubation was then carried out with either 10 nM testosterone or 10 µM of each of the peptides or with vehicle only (control). The medium was removed by aspiration after 30 min, and cells were fixed for 15 min in absolute methanol containing DAPI (4′,6-diamidino-2-phenylindole). After aspirating the fixative, cells were treated with blocking solution (3% BSA and 0.3% Triton X-100 in PBS) for 1 h. This was then replaced by PBS containing 1% BSA, 0.3% Triton X-100, and the primary antibody against phospho-ERK1/2 or phospho-CREB, claudin-1, or claudin-5 (Cell Signaling Technology).

For visualization by immunofluorescence, the medium containing the primary antibody was replaced with PBS containing 1% BSA, 0.3% Triton X-100, and the secondary antibody (goat anti-rabbit IgG diluted at 1:500 in 2% FCS, 0.1 Triton X-100 in PBS) labeled with AlexaFluor 488 (Life Technologies, Darmstadt, Germany). This medium was removed by aspiration after 1 h at room temperature and cells were washed in PBS 3 times. All incubation steps with UV-sensitive reagents were carried out in the dark. An inverse Olympus IX81 microscope equipped with the corresponding fluorescence system (Olympus, Hamburg, Germany) was used to acquire images.

Fluorescence was measured according to the instructions of the ImageJ software program (freely available at http://rsbweb.nih.gov/ij/, accessed on 23 December 2021) according to the protocol provided (http://www.slu.se/PageFiles/388774/Pacho%20ImageJ¬¬%20-measuring-cell-fluo¬rescence.pdf, accessed on 23 December 2021). Only green fluorescence was considered. The fluorescence of 3 × 25 cells closest to the diagonals of photomicrographs from 3 independent experiments was considered for statistical analysis. The software program GraphPad Prism4 (GraphPad Software, Inc., La Jolla, CA, USA) was used for data point analysis. All antibodies used for the immunofluorescence experiments and their concentrations (dilutions) are summarized in Supplementary Table S1.

### 2.6. Cell-Surface Labeling with Testosterone-BSA-FITC

A total of 93 RS2 cells were cultured at a density of 5 × 10^3^ cells per well in 24-well plates as described above. After reaching about 80% confluence, the cells were treated with either 10 nM testosterone or 1 µM of each of the peptides for 1 h. Both peptide-treated cells and controls were also treated with ethanol vehicle at the same concentration as other plates of cells treated with testosterone dissolved in ethanol. Testosterone 3-(O-carboxymethyl)oxime:BSA-fluorescein isothiocyanate (testosterone-BSA-FITC) conjugate (Sigma-Aldrich, Munich, Germany) was diluted in Tris-Buffer (pH 7.2) and then added to each well at a final concentration of 5 µM. After incubation for an additional 20 min at room temperature, the medium was aspirated and cells were fixed in 400 µL ice-cold methanol containing 20 ng of 4,6-diamino-2-phenylindole (DAPI) at room temperature. This solution was aspirated after 15 min and cells were washed with 700 μL PBS. Cell imaging was carried out in 400 µL PBS using an inverse Olympus IX81 microscope (Olympus, Hamburg, Germany).

In order to account for non-specific binding, cells were incubated for 20 min with 5 µM BSA-FITC (lacking the testosterone moiety) diluted in Tris-Buffer (pH 7.2) at room temperature. After washing twice with PBS and fixing in absolute methanol containing DAPI for 15 min, images were obtained as described above.

To prove that ZIP9 is the sole membrane-bound androgen receptor, 93RS2 cells that had been treated with either ZIP9-specific siRNA (ZIP9-siRNA) or negative-control siRNA (nc-siRNA) for 24 h (see next paragraph) were washed in 700 μL PBS and then incubated with testosterone-BSA-FITC as described above. Images were taken as described above after incubation of the cells in methanol containing DAPI to label the nuclei.

### 2.7. Silencing ZIP9 Expression by siRNA

A total of 6 × 10^4^ 93RS2 cells were cultured in 2-cm wells of a 6-well plate for 24 h. Thereafter, cells were treated for 48 h either with ZIP9-specific siRNA (ZIP9-siRNA; 5′GGAUUAAGUAAGAGCAGUAtt3′ and 5′UACUGCUCUUACUUAAUCCta3′) to silence ZIP9 expression or with an appropriate negative control siRNA (nc-siRNA), as described previously [6]. ZIP9-siRNA and nc-siRNA were part of the kit Silencer^®^ Select Pre-designed siRNA, provided by Invitrogen (Thermo Fisher Scientific, Schwerte, Germany). After the siRNA treatment, RNeasy^®^ Mini Kit (Qiagen GmbH, Hilden, Germany) was used to isolate mRNA. The concentration of the extracted mRNA was quantified by the NanodropTM One spectrophotometer (Thermo Fisher Scientific, Schwerte, Germany). Samples were measured in duplicate and the average values were used.

### 2.8. Reverse Transcription PCR (RT-PCR)

RT-PCR was applied in order to assess the success of silencing ZIP9 expression by siRNA. mRNA that had been isolated from cells treated with either nc-siRNA or ZIP9-specific siRNA was processed to cDNA using the Reverse Transcription System (Promega GmbH, Mannheim, Germany) according to the protocol of the provider. A total of 1 µg cDNA, quantified by the NanodropTM One spectrophotometer, was used in the PCR-Master-Mix ready-to-load (Bio&SELL GmbH, Feucht, Germany) to detect ZIP9-specific cDNA. As primers were used the oligonucleotides 5′GCTGCATGCCTACATTGGTG3′ and 5′ GTTAGTGCTGGTGTCCTCAGGG3′, which amplify a ZIP9-specific cDNA fragment of 502 bp. As a control, the rat housekeeping gene GAPDH was amplified in parallel using the oligonucleotide primers 5′GACCCCTTCATTGACCTCAAC3′ and 5′GATGACCTTGCCCACAGCCTT3′, which amplify a GAPDH-specific fragment of 561 bp. All primers were from Invitrogen by Thermo Fisher Scientific.

### 2.9. Transepithelial Electrical Resistance (TER)

A total of 3 × 10^4^ cells were added to 0.4-µm pore ThinCertsTM (Greiner Bio-one GmbH, Frickenhausen, Germany) arranged within 1-cm wells of a 24-well plate. Cells were cultured for 24 h. Stimulation was then carried out with either testosterone or peptides at concentrations of 0.1, 1, 10, or 100 nM (testosterone) or 0.1, 1, 10, or 100 µM (peptides). Control incubations received ethanol vehicle only. TER was determined after 0, 24, 48, and 72 h, with the first measurement being made immediately after addition of testosterone or peptides. Stimulants remained in the cell medium for the entire duration of the experiment, and during this time the medium was not renewed. A Millicell ERS-2 epithelial Volt-Ohm Meter (Merck Millipore, Darmstadt, Germany) was used to measure TER. Each experiment was repeated three times. Similar experiments were carried out after silencing expression of ZIP9 by siRNA.

### 2.10. Statistical Analysis

Data were analyzed by GraphPad Prism4 software and by applying either the unpaired *t*-test with Welch’s corrections or one-way ANOVA with repeated measures and Dunnett´s comparison of all data to the control. Significance was accepted at *p* ≤ 0.05.

## 3. Results

### 3.1. Silencing ZIP9 Expression by siRNA

**Figure 1:**
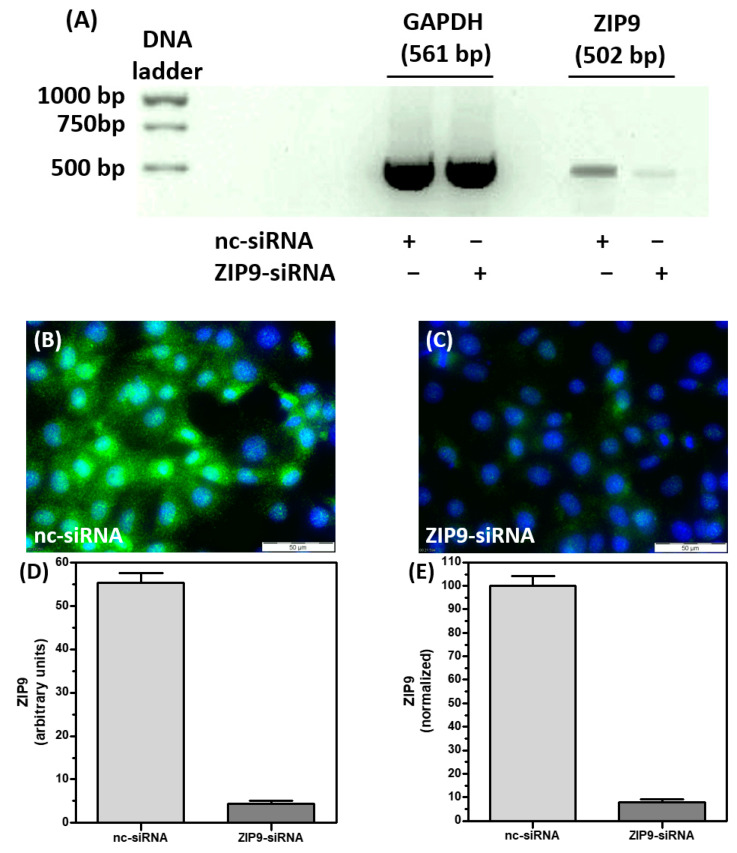
Immunofluorescence of ZIP9 before and after ZIP9 knock down in Sertoli cells. The 93RS2 cells were treated with either ZIP9-specific siRNA (ZIP9-siRNA) or negative control siRNA (nc-siRNA). (**A**) PCR showing the ZIP9-specific amplificate before or after silencing ZIP9 expression. The GAPDH–specific amplificate served as control. (**B**,**C**) Detection of ZIP9 protein by immunofluorescence before and after silencing ZIP9-specific mRNA expression. Green fluorescence corresponds to ZIP9, nuclei are stained blue. (**D**,**E**) Statistical analysis of ZIP9-specific immunofluorescence (n = 30; means ± SEM).

The direct involvement of ZIP9 in the cellular responses described in the following paragraphs was addressed by investigating the effect of silencing its expression by siRNA. It was important to first confirm the successful knockdown of ZIP9 expression by RT-PCR and immunofluorescence. Figure 1A shows that in comparison with nc-siRNA-treated cells, the presence of ZIP9-specific mRNA/cDNA in 93RS2 cells that had been treated with ZIP9-specific siRNA was greatly reduced. This reduction was also reflected in the significant reduction of ZIP9 protein expression in cells that were treated with ZIP9-siRNA. In these cells green fluorescence corresponding to the presence of ZIP9 protein accounted only for 7.93 ± 1.1% of the ZIP9-specific fluorescence under control conditions (Figure 1B–E).

### 3.2. Tetrapeptides Target the Androgen-Binding Site of ZIP9

**Figure 2:**
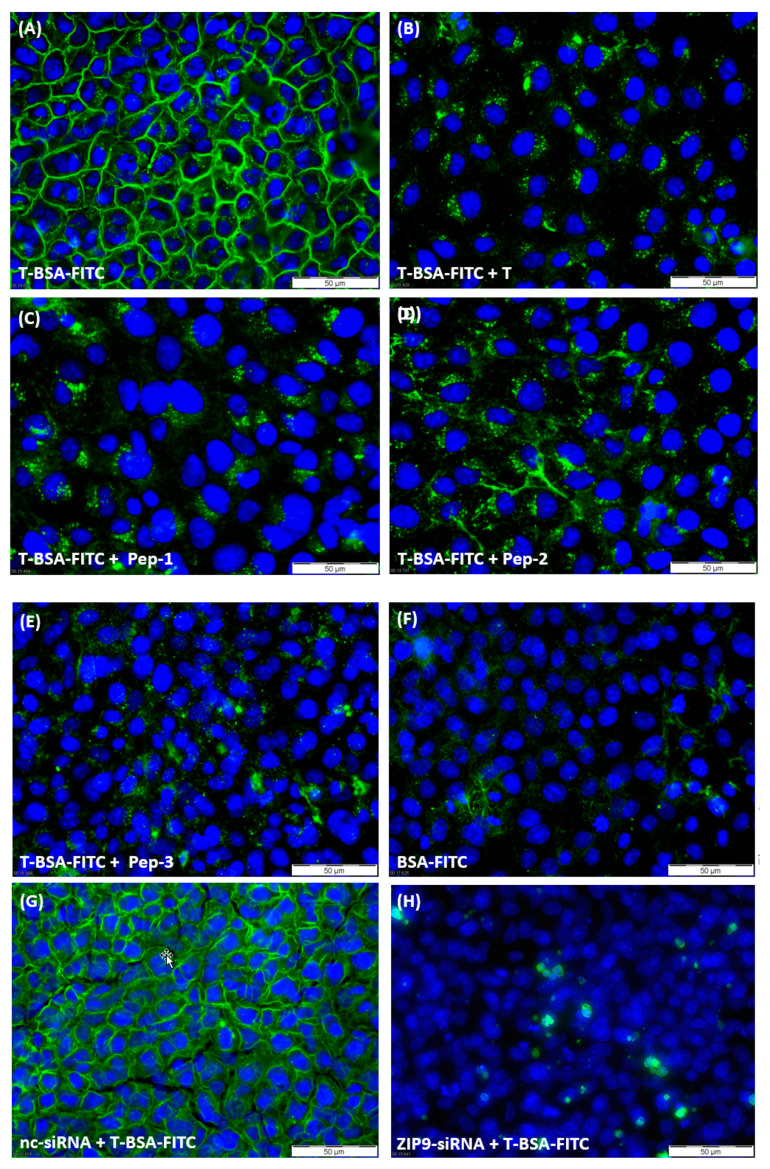
Testosterone-BSA-FITC (T-BSA-FITC) membrane labelling of 93RS2 cells. T-BSA-FITC or BSA-FITC are visualized as green fluorescence, nuclei are stained blue. (**A**) 93RS2 cells were treated with T-BSA-FITC, which leads to distinct membrane labelling. (**B**–**E**) Testosterone (10 nM) or peptides 1–3 (10 µM) were added to cells treated with T-BSA-FITC. (**F**) Labelling of 93RS2 cells with BSA-FITC lacking the T moiety. (**G**) The 93RS2 cells were treated with nc-siRNA prior to T-BSA-FITC incubation. (**H**) Cells were incubated with ZIP9-specific siRNA and then treated with T-BSA-FITC.

Testosterone-BSA-FITC (T-BSA-FITC) is a testosterone analogue that does not penetrate the plasma membrane [7,24,25,26]; therefore, it is suitable to detect plasma membrane-bound androgen receptors bearing an extracellularly accessible androgen binding site. Incubation of 93RS2 cells with T-BSA-FITC led to uniform green labeling of the extracellular surface of the cells, supporting the previously calculated 3D model of ZIP9 that identifies its androgen binding site on its extracellular surface (Figure 2A). When testosterone was included in the incubation medium (Figure 2B) or any of the peptides (Figure 2C–E), membrane labeling by T-BSA-FITC was greatly suppressed. Relatively low signals of green fluorescence were also obtained when cells were incubated with BSA-FITC (lacking the testosterone moiety; Figure 2F) indicating that this labeling and the residual labeling in Figure 2B–E is due to non-specific interactions of the fluorescent compounds with some components of the plasma membrane or the cytosol.

To ensure that T-BSA-FITC or the peptides are specifically targeting the ZIP9 protein and not some other membrane component that might also be capable of binding androgens, labeling experiments were repeated after silencing the expression of ZIP9 by siRNA.

Figure 2G shows that the treatment of the cells with nc-siRNA did not affect the labeling of the cell membranes by testosterone-BSA-FITC (compare with Figure 2A). When expression of ZIP9 was suppressed by ZIP9-specific siRNA, however, labeling of the cell membranes was completely prevented and only some cytosolic, non-specific labeling was observed in a few cells (Figure 2H).

### 3.3. Tetrapeptides Trigger Phosphorylation of Erk1/2 and CREB/ATF-1

In the so-called non-classical signaling pathway of testosterone, phosphorylation (activation) of Erk1/2 by the steroid hormone leads to the phosphorylation (activation) of transcription factors such as CREB or ATF-1 and to the stimulation of the expression of cell-specific proteins. The mediator of these effects in 93RS2 Sertoli cells is ZIP9 [5,6,7].

**Figure 3:**
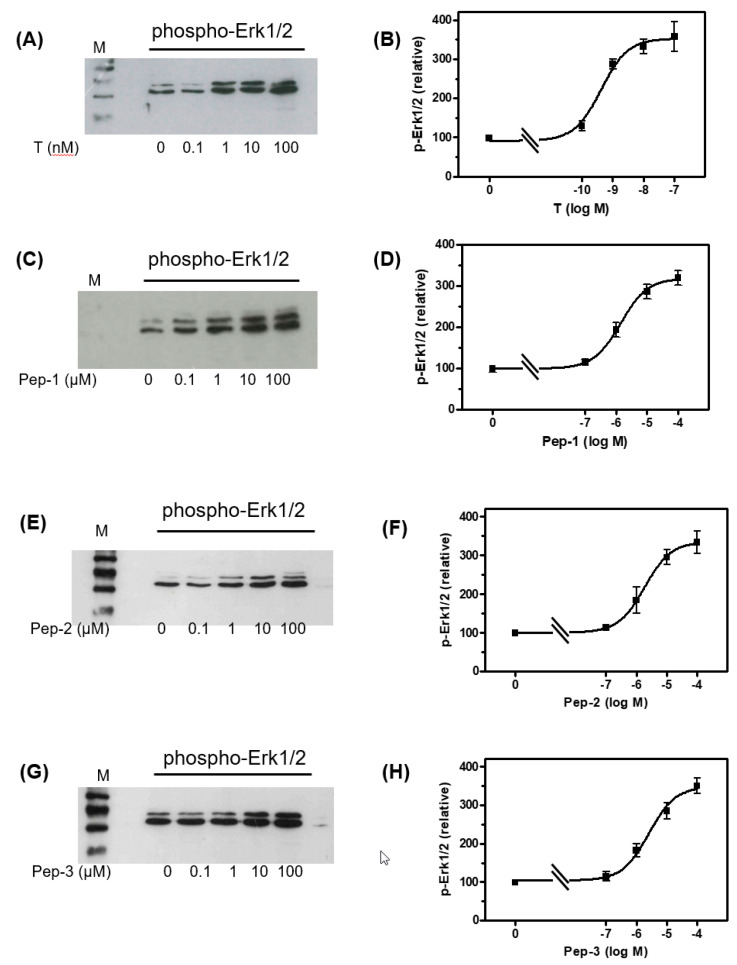
Western blot of phospho-Erk1/2 in Sertoli cells. The 93RS2 cells were stimulated for 30 min with various concentrations of testosterone or peptides. (**A**) Phospho-Erk1/2 detection in Western blot after stimulation with T, (**C**) after stimulation with Pep-1, (**E**) after stimulation with Pep-2 or (**G**) after stimulation with Pep-3. (**B**) The semi-logarithmic plot of relative p-Erk1/2 signals reveal for T an EC_50_ = 0.42 ± 1.54 nM, (**D**) for Pep-1 an EC_50_ = 1.39 ± 1.36 µM, (**F**) for Pep-2 an EC_50_ = 2.59 ± 1.05 µM and (**H**) for Pep-3 an EC_50_ = 1.87 ± 1.6 µM. For each diagram: n = 3; means ± SD.

To determine whether the peptides trigger effects similar to those of testosterone, Western blots (Supplementary Figure S1) were carried out to detect phospho-Erk1/2 in lysates from cells that had been incubated for 30 min with various concentrations of the peptides or testosterone. All peptides and testosterone stimulated in a concentration-dependent manner the phosphorylation of Erk1/2 within 30 min (Figure 3). The expression of total Erk1/2 in lysates from cells treated with testosterone or any of the peptides was also stimulated (not shown). Testosterone stimulated the phosphorylation of Erk1/2 with an EC_50_ = 0.42 ± 1.54 nM, Pep-1 with an EC_50_ = 1.39 ± 1.36 µM, Pep-2 with an EC_50_ = 2.59 ± 1.05 µM and Pep-3 with an EC_50_ = 1.87 ± 1.6 µM.

**Figure 4:**
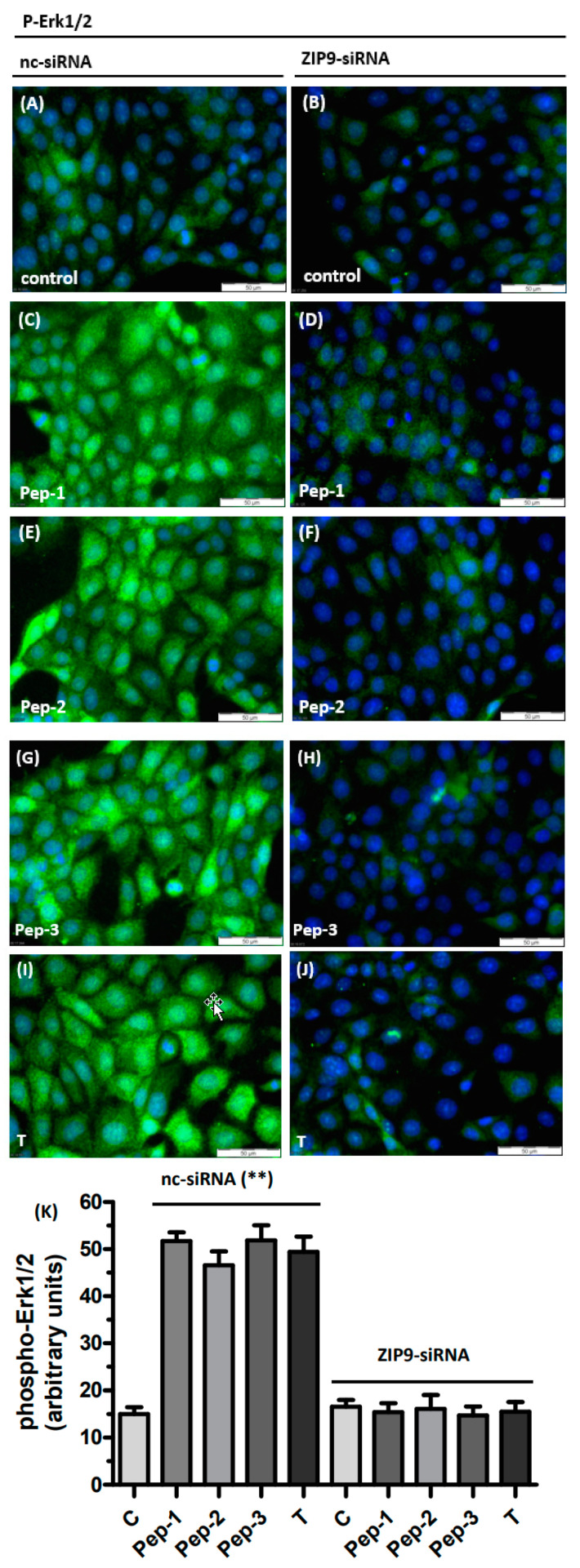
Detection of phospho-Erk1/2 in Sertoli cells by immunofluorescence. Incubation of 93RS2 cells with 10 nM of T or with 10 µM of each of the peptides occurred for 30 min. Nuclei are stained blue, phospho-Erk1/2 is represented by green fluorescence. Cells were treated either with negative control siRNA (**A**,**C**,**E**,**G**,**I**) or with ZIP9-directed siRNA (**B**,**D**,**F**,**H**,**J**). (**A**) Phospho-Erk1/2 in unstimulated Sertoli cells. (**C**) Phospho-Erk1/2 in cells treated with Pep-1, (**E**) treated with Pep-2, (**G**) treated with Pep-3 and (**I**) treated with T. When ZIP9 expression is silenced by siRNA peptides or T fail to stimulate Erk1/2 phosphorylation (**B**,**D**,**F**,**H**,**J**). (**K**) Statistical analysis of green fluorescence shown in panels A-J (n = 3 × 25; means ± SEM; ** *p* ≤ 0.01).

These findings were confirmed on a cellular basis using immunofluorescence. Cells were incubated with 10 nM testosterone or 10 µM of each of the peptides for 30 min and then subjected to a fixation/immunostaining procedure as described under “Methods” in order to detect the phosphorylated forms of Erk1/2 by immunofluorescence. In the absence of any stimulus, phospho-Erk1/2, indicated by the green fluorescence, was rather modest (Figure 4A). Addition of either of the peptides or testosterone triggered the phosphorylation of Erk1/2, which spread as green fluorescence over the entire area of each of the 93RS2 cells that had been treated with nc-siRNA (Figure 4C,E,G,I). Silencing ZIP9 expression abolished the stimulation of Erk1/2 phosphorylation by either of the peptides or testosterone (Figure 4D,F,H,J). The level of phosphorylation under these conditions was the same as in nc-siRNA-treated controls (Figure 4K).

**Figure 5:**
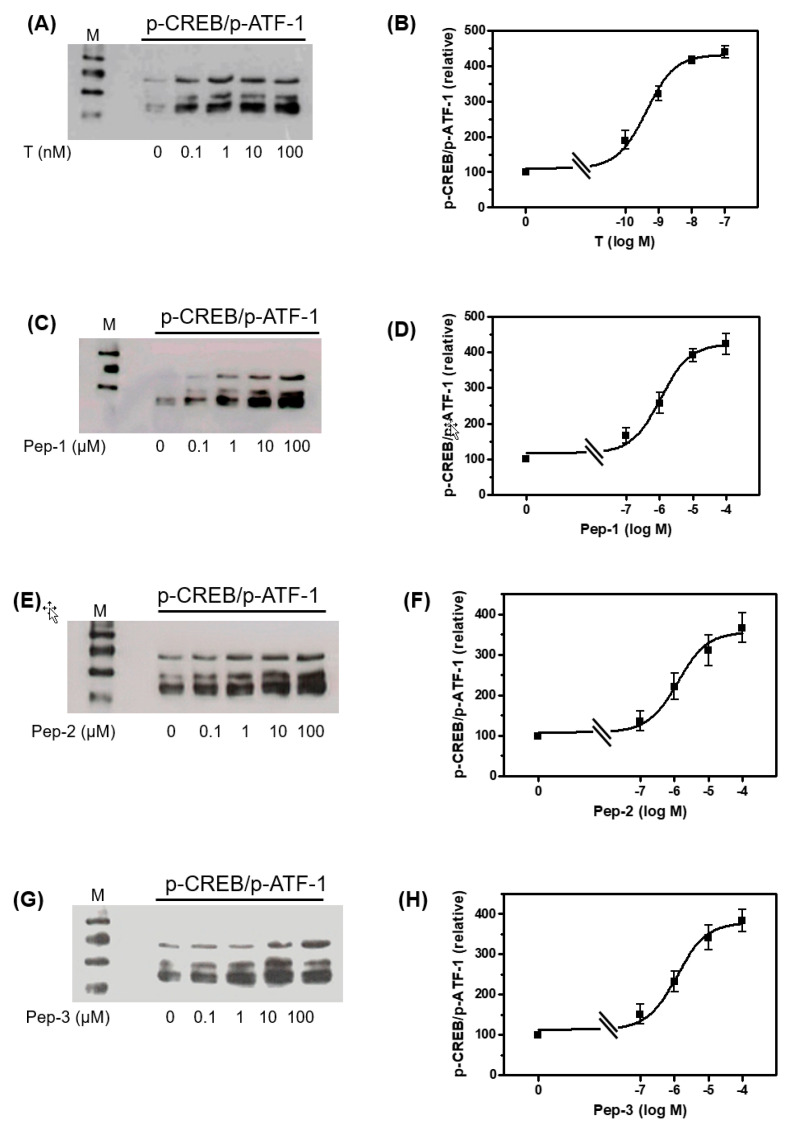
Western blot of phospho-CREB and phospho-ATF-1 in Sertoli cells. The 93RS2 cells were stimulated for 30 min with various concentrations of testosterone or peptides. Phospho-CREB is represented by the band at around 45 kDa, phospho-ATF-1 by the two bands at 38 and 34 kDa. (**A**,**C**,**E**,**G**) p-CREB/p-ATF-1 detection after stimulation with T, Pep-1, Pep-2 or Pep-3. (**B**,**D**,**F**,**H**) Semi-logarithmic plots of relative p-CREB/p-ATF-1 signals reveal for T an EC_50_ = 0.43 ± 1.33 nM, for Pep-1 an EC_50_ = 1.09 ± 1.44 µM, for Pep-2 an EC_50_ = 1.27 ± 1.79 µM and for Pep-3 an EC_50_ = 1.21 ± 1.56 µM. For each diagram: n = 3; means ± SD.

The Western blots shown in Supplementary Figure S2 and in Figure 5 demonstrate that peptides and testosterone also stimulate the phosphorylation of the transcription factors CREB and ATF-1. The two related proteins share identical phosphorylation sites (Ser133 and Ser63, respectively) that cannot be distinguished by the antibody used [27]. Nevertheless, p-CREB is represented by the band at around 45 kDa and p-ATF-1 by the two bands at 38 and 34 kDa [28]. Testosterone or peptides stimulated CREB/ATF-1 phosphorylation with EC_50_ values similar to the those obtained for the stimulation of Erk1/2 phosphorylation. Thus, testosterone stimulated CREB/ATF-1 phosphorylation with an EC_50_ = 0.43 ± 1.33 nM, Pep-1 with an EC_50_ = 1.09 ± 1.44 µM, Pep-2 with an EC_50_ = 1.27 ± 1.79 µM, and Pep-3 with an EC_50_ = 1.21 ± 1.56 µM.

The EC_50_ values were within the same range, independent of whether the densitometric analysis of the Western blots were carried out for p-CREB- or p-ATF-1-bands separately or if all three bands in each lane are treated as a single value. In Figure 5, bands representing p-CREB and p-ATF-1 were analyzed as one.

Maximum stimulation of Erk1/2 of CREB/ATF-1 phosphorylation was obtained between 10 and 100 nM for testosterone, or between 10 and 100 µM for each of the peptides. For that reason, all of the following experiments were carried out at either 10 nM testosterone or 10 µM of each of the peptides.

**Figure 6:**
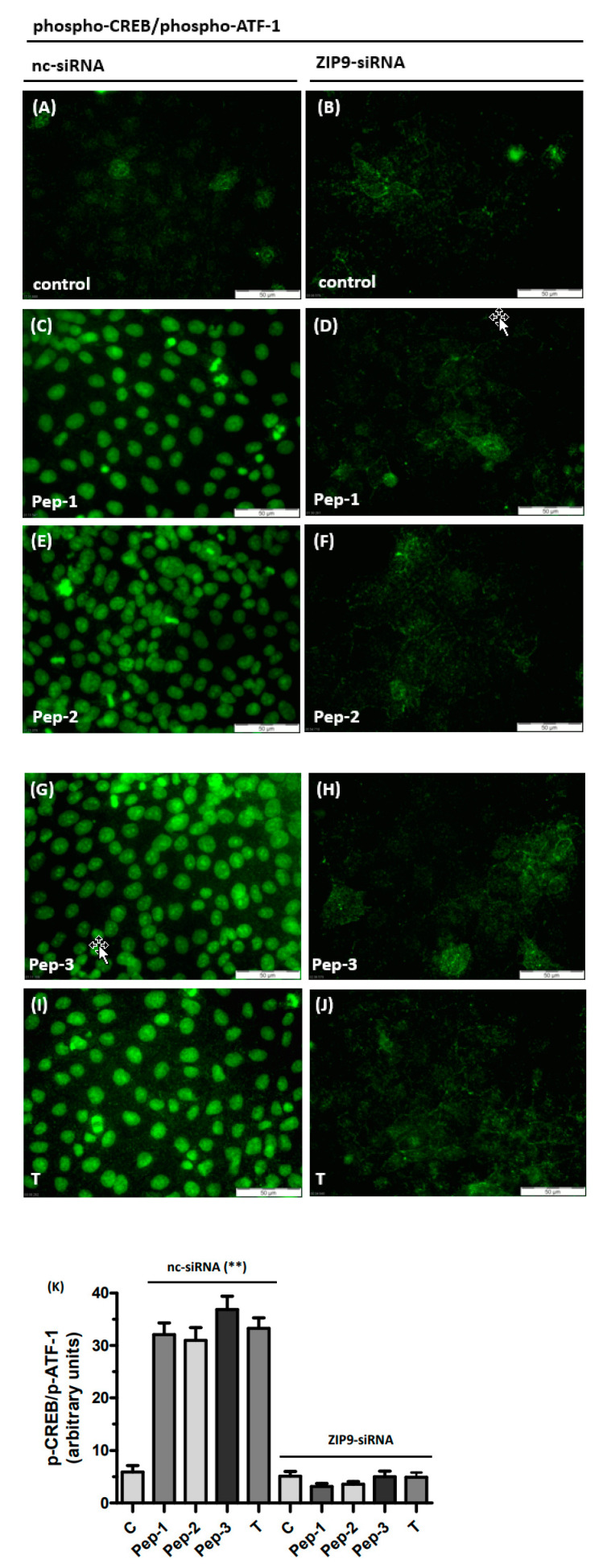
Detection of phospho-CREB/phospho-ATF-1 in Sertoli cells by immunofluorescence. The 93RS2 cells were treated with 10 nM of T or 10 µM of each of the peptides for 30 min. Nuclei are stained blue, the two transcription factors are represented by the green fluorescence. Cells were treated either with negative control siRNA (**A**,**C**,**E**,**G**,**I**) or with ZIP9-directed siRNA (**B**,**D**,**F**,**H**,**J**). (**A**) Phospho-CREB/phospho-ATF-1 in control cells. (**C**) Phospho-CREB/phospho-ATF-1 in cells treated with Pep-1, (**E**) in cells treated with Pep-2, (**G**) in cells treated with Pep-3 and (**I**) in cells treated with T. When ZIP9 expression is silenced by siRNA peptides or T fail to stimulate Erk1/2 phosphorylation(**B**,**D**,**F**,**H**,**J**). (**K**) Statistical analysis of green fluorescence shown in panels A-J (n = 3 × 25; means ± SEM; ** *p* ≤ 0.01).

CREB or ATF-1 are transcription factors and should therefore be detectable within the nuclei by immunofluorescence. Figure 6 shows that treatment with testosterone or any of the peptides stimulated phosphorylation of the transcription factors within the nuclei (Figure 6C,E,G,I). Green fluorescence representing phosphorylated CREB or ATF-1 was largely absent in nuclei of untreated controls (Figure 6A,B) or in cells in which ZIP9 expression was prevented by ZIP9-siRNA (Figure 6D,F,H,J). Under these latter conditions, the level of phosphorylation is about sevenfold lower than that observed in stimulated cells expressing ZIP9 (Figure 6K).

### 3.4. Stimulation of the Expression of Proteins Involved in Tight Junction Formation

**Figure 7:**
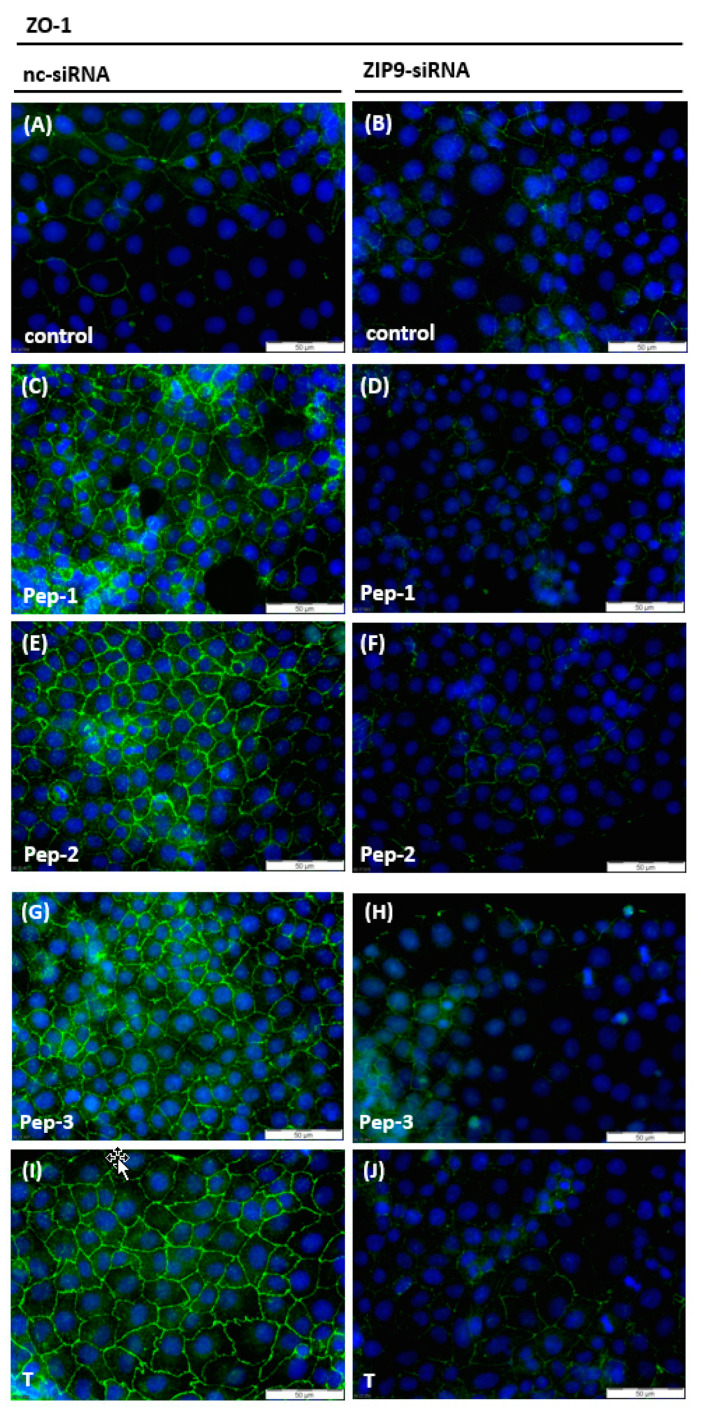
Detection of ZO-1 in Sertoli cells by immunofluorescence. Nuclei are stained blue, ZO-1 is visible as green fluorescence. (**A**,**B**) Unstimulated 93RS2 cells treated with nc-siRNA or ZIP9-specific siRNA show basal level of ZO-1 expression. (**C**,**E**,**G**,**I**) Cells that are treated with nc-siRNA and stimulated with peptides (10 µM) or testosterone (10 nM) for 24 h show increased staining of ZO-1 at their plasma membranes. (**D**,**F**,**H**,**J**) Cells treated with ZIP9-siRNA did not respond to peptide or testosterone treatment by increasing ZO-1 expression.

Formation, maintenance, and dynamics of the BTB are regulated by testosterone-induced signaling events that include activation of Erk1/2 and CREB and lead to the stimulation of the expression of proteins that participate in the formation of TJs at the BTB [29,30,31]. This also applies for 93RS2 Sertoli cells [6]. In 93RS2 cells that had been treated with nc-siRNA, all peptides and testosterone stimulated the expression of zona occludens (ZO-1), a cytosolic protein associated with TJs [32] including those formed between Sertoli cells at the BTB [33] (Figure 7C,E,G,I). Some faint green basal fluorescence was visible between some neighboring cells in untreated control cultures (Figure 7A,B) and between cells that had been treated with ZIP9-siRNA before exposure to peptides or testosterone (Figure 7D,F,H,J).

**Figure 8:**
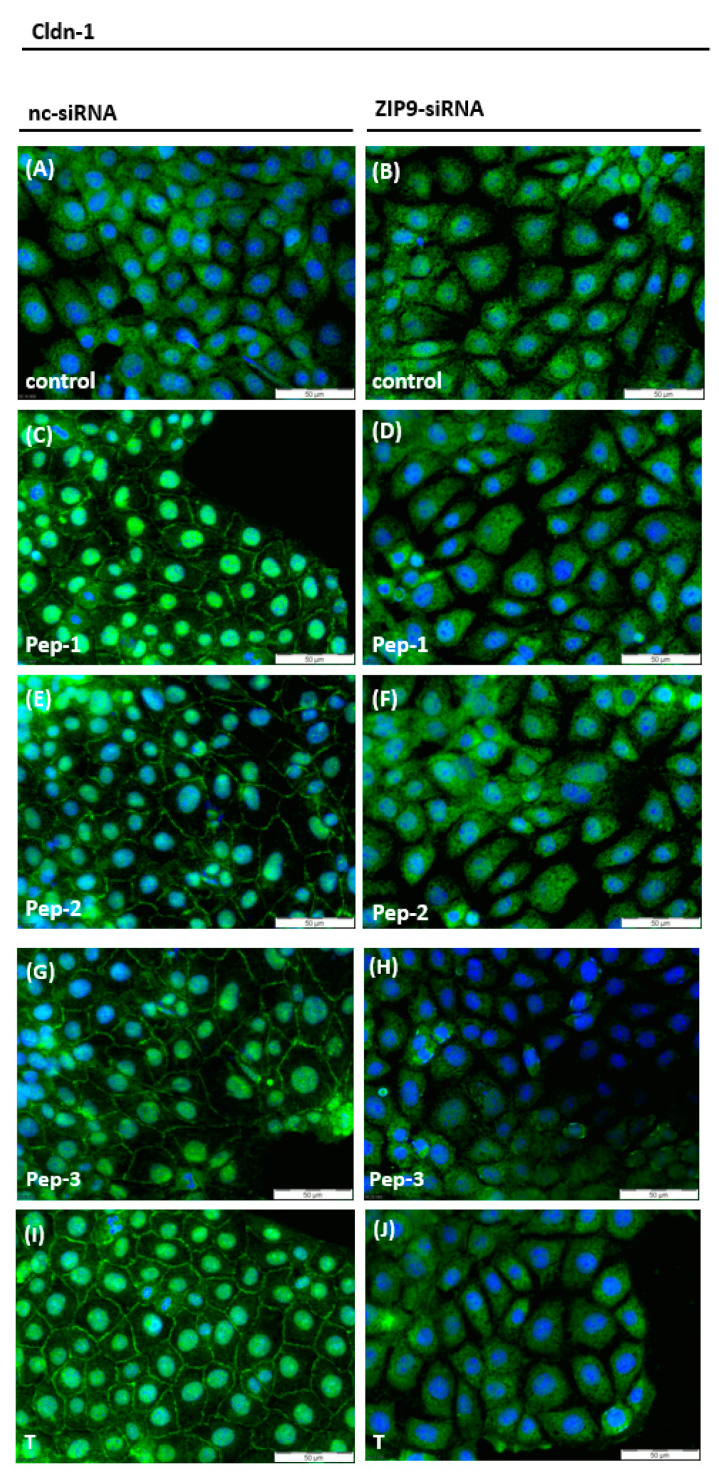
Detection of Claudin-1 in Sertoli cells by immunofluorescence. Nuclei are stained blue, Claudin-1 is represented by green fluorescence. (**A**,**B**) Control cells without stimulation before and after ZIP9 knock down. Claudin-1 is evenly distributed in the cytosol. (**C**,**E**,**G**,**I**) Cells were treated with nc-siRNA and stimulated with peptides (10 µM) or T (10 nM) for 24 h. Claudin-1 is visible within the nuclei and also in plasma membranes. (**D**,**F**,**H**,**J**) show 93RS2 cells treated with ZIP9-siRNA before exposure to peptides or T. Claudin-1 is evenly distributed within the cytosol.

Testosterone has been shown to stimulate claudin-1 and claudin-5 expression in rodent Sertoli cells [6,29,34,35], although peptides and testosterone do not seem to influence the expression level of claudin-1 but rather its distribution and localization. As shown in Figure 8A, the green fluorescence that corresponds to claudin-1 was uniformly distributed within the cytosol under basal conditions. Treatment with either of peptides or testosterone triggered re-distribution of claudin-1 from the cytosol to the membranes or to the nuclei of 93RS2 cells, which appeared bright green (Figure 8C,E,G,I). This re-distribution was not observed when the expression of ZIP9 was prevented by ZIP9-siRNA (Figure 8D,F,H, J). Under these conditions, claudin-1 protein was uniformly distributed in the cytosol, as in the untreated controls (Figure 8A,B).

**Figure 9:**
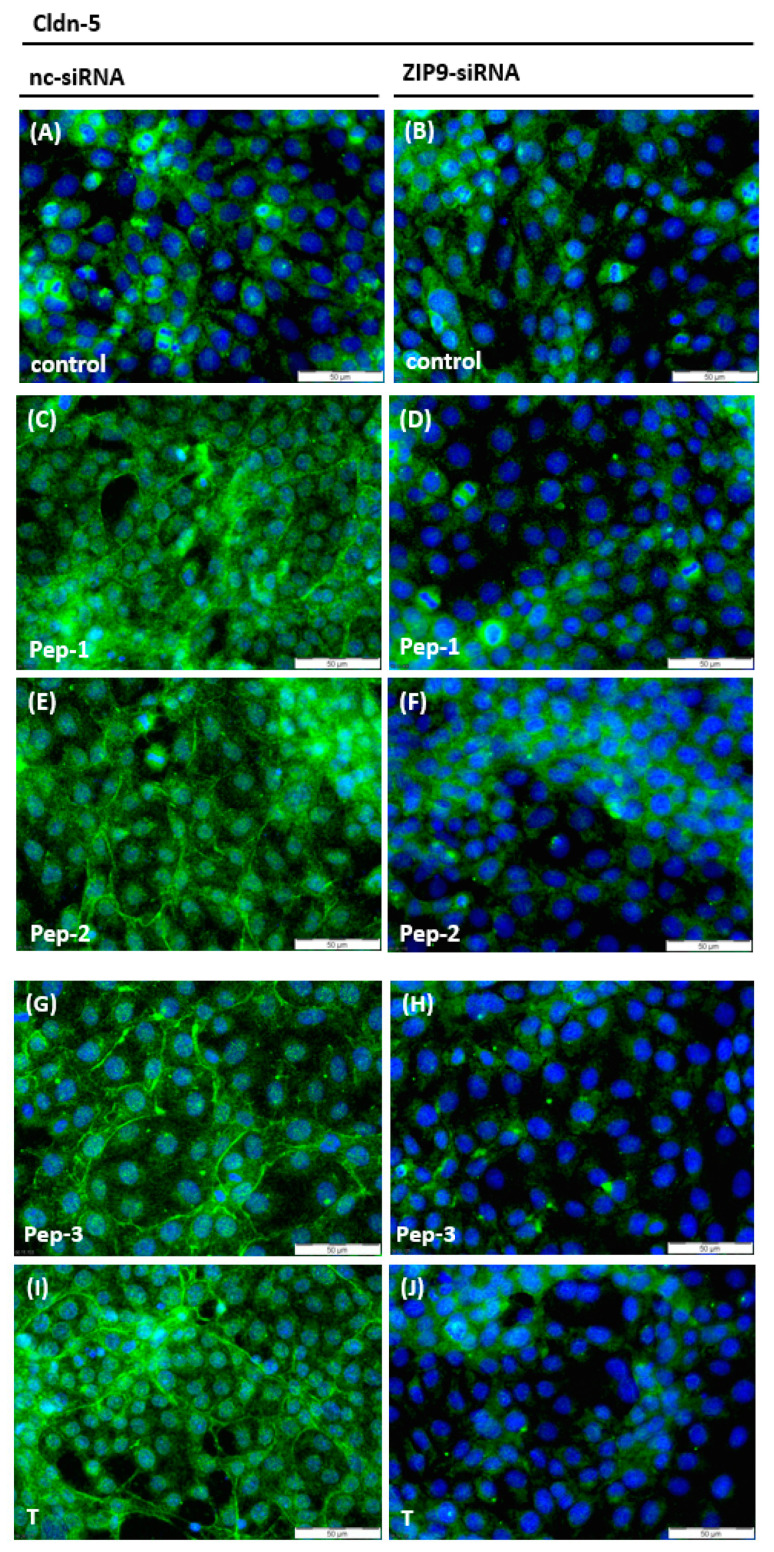
Detection of Claudin-5 in Sertoli cells by immunofluorescence. Claudin-5 is represented by green fluorescence, nuclei are stained blue. (**A**) Detection of basal levels of Claudin-5 in 93RS2 cells treated with nc-siRNA, without any further stimulation. (**B**) Bassal levels of Claudin-5 in cells treated with ZIP9-siRNA, without any further treatment. (**C**,**E**,**G**,**I**) Cells treated with nc-siRNA and stimulated with peptides (10 µM) or testosterone (10 nM) for 24 h. Claudin-5 expression is increased in the cytoplasm and visible in the membranes. Cells shown in (**D**,**F**,**H**,**J**) were treated with ZIP9-siRNA before exposed to peptides or testosterone. Only basal levels of Claudin-5 are detected.

Peptides and testosterone stimulated the expression of claudin-5, which was present not only in the cytosol, as observed in control cultures (Figure 9A), but to a great extent in the plasma membranes of the 93RS2 Sertoli cells (Figure 9C,E,G,I). After knockout of ZIP9 expression (Figure 9D,F,H,J), the expression level and localization of claudin-5 resembled the situation in the two control cultures (Figure 9A,B).

### 3.5. Stimulation of Tight Junction Formation by the Peptides and Testosterone

**Figure 10:**
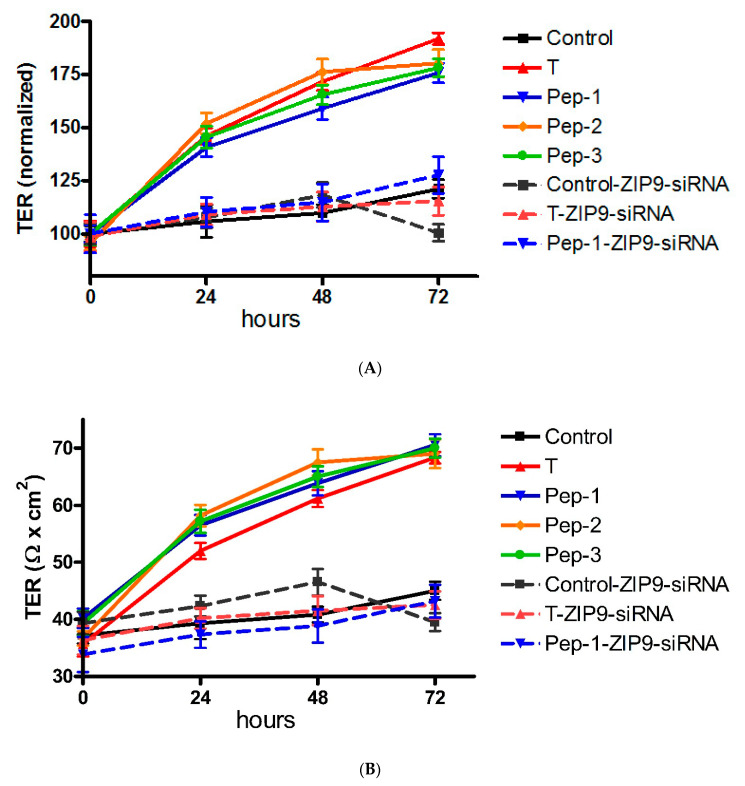
Transepithelial electrical resistance across Sertoli cell layers. The 93RS2 cells were treated with either nc-siRNA or ZIP9-siRNA before exposed to peptides (10 µM) or testosterone (10 nM). IAPG = Pep-1; GVSG = Pep-2; GVVG = Pep-3. (**A**) Measurements of TER were taken after 0, 24, 48 and 72 h. (**B**) Normalization of results shown in (**A**). For each data point: n = 6; means ± SEM).

The possibility that TJ formation is increased between 93RS2 Sertoli cells by the peptides or testosterone was investigated by measuring TER across cell monolayers. As can be seen in Figure 10A, TER increased from 34.2 to 42 Ω × cm^2^ over the course of 3 days in the absence of peptides or testosterone. In the presence of peptides or testosterone, TER was elevated by 70% to 90% above control values after 3 days (Figure 10B). When ZIP9 expression was prevented by siRNA, testosterone and the three peptides failed to stimulate TER increase under otherwise same conditions (Figure 10A,B).

## 4. Discussion

In the investigation presented here, three tetrapeptides that were designed to fit within the androgen binding site of ZIP9 were tested for their ability to induce effects associated with TJ formation in the rat testis-derived Sertoli cell line 93RS2. This cell line, which lacks the classical AR, expresses ZIP9 and is therefore suitable for addressing androgenic effects that are generated solely through this cell-surface receptor.

The ability of the peptides to bind to the androgen binding site of ZIP9 was addressed by looking at their ability to displace testosterone-BSA-FITC binding to the cells. All peptides, as well as testosterone, were able to prevent the binding of the testosterone analogue, which supports the 3D model predicting an extracellular localization of the androgen binding site [20] and qualifies the peptides as ZIP9 androgen binding site-specific probes. This latter conclusion was further verified by demonstrating the complete loss of testosterone-BSA-FITC labeling from the 93RS2 cell surface after silencing ZIP9 expression by siRNA.

Erk1/2 constitutes a critical component of the so-called non-classical pathway of testosterone. Activation of this kinase within the Src/c-Raf/Erk1/2/CREB signaling cascade by testosterone in Sertoli or germ cells is required for the survival of spermatocytes and the production of mature spermatozoa [36,37,38,39]. It was therefore reasonable to investigate whether any of the peptides are able to activate Erk1/2 in 93RS2 Sertoli cells and to compare their actions with the actions of testosterone. As anticipated from earlier findings [6], testosterone stimulated the phosphorylation of Erk1/2 within 30 min without affecting the expression of the enzyme protein. The EC_50_ value for the effect under these conditions was about 0.5 nM. Each of the peptides also triggered rapid stimulation of Erk1/2 phosphorylation in a concentration-dependent manner with EC_50_ values in the range of 1.5 µM.

Further experiments showed that activation of Erk1/2 phosphorylation by testosterone and the peptide analogues depends on the presence of ZIP9. Thus, stimulation of Erk1/2 phosphorylation by testosterone or any of the peptides did not occur if ZIP9 expression was silenced with siRNA, indicating that in this cell type ZIP9 is the sole conductor of androgenic effects and the initiation point from which the so-called non-classical signaling pathway of testosterone originates.

In this signaling pathway, Erk1/2 activation leads to the activation of the transcription factors CREB and ATF-1 by phosphorylation. All peptides tested here acted the same as testosterone and induced phosphorylation of CREB and ATF-1 in a concentration-dependent manner within 30 min. CREB and ATF-1 belong to the family of bZip (basic domain leucine zipper) transcription factors. Although they share little similarity at level of the primary amino acid sequence, they have similar functional domains in their structural organization, including—in addition to the bZIP region or dimerization domains—phosphorylation (P-box) domains [40]. For this reason, the antibody used in the present study, although declared to be p-CREB specific, also detected p-ATF-1. Testosterone stimulated CREB and ATF-1 phosphorylation with an EC_50_ of about 0.5 nM, and each of the peptides had EC_50_ values within the range of 1 µM. These values are consistent with the EC_50_ values obtained for the stimulation of Erk1/2 phosphorylation by testosterone or each of the peptides. Here, too, maximum effects were obtained at 10 nM testosterone and 10 µM for any of the peptides. Stimulation of CREB or ATF-1 phosphorylation was also shown to depend on the presence of ZIP9: silencing its expression by siRNA led to the complete loss of the stimulatory effect of testosterone or peptides on the phosphorylation of the two transcription factors, as demonstrated by immunofluorescence experiments.

These findings concerning the stimulation of Erk1/2 or CREB/ATF-1 phosphorylation are consistent with the conclusion that all three peptides tested act as pro-androgens and that none of them is an anti-androgen. This was not necessarily expected. Interestingly, although pro-androgenic, all three peptides induce their testosterone-like effects at higher EC_50_ values than the original substrate testosterone.

Longer incubation with testosterone or peptides was found to influence the dynamics of TJs formed between neighboring 93RS2 Sertoli cells. Expression of ZO-1 and claudin-5 was increased, whereas expression of claudin-1 did not seem to be affected. Importantly, peptides and testosterone affected the localization and distribution of the latter protein, which upon stimulation moved from the cytosol to the nucleus and to the plasma membrane. Testosterone-induced re-distribution of claudins has been observed previously [30,41,42].

Claudins and ZO-1 are essential for the formation of TJs and for the maintenance of the BTB, which functions as a physical and immunological barrier that regulates the paracellular flow of water, electrolytes, nutrients, hormones, and other substances from and to the gonads and at the same time protects the meiotic and post-meiotic stages of germ cells from cells of the immune system [43]. Maintenance and protection of the BTB are absolutely critical for male fertility [43,44]. BTB defects are associated with impaired spermatogenesis [45,46], and BTB integrity is associated with testicular dysgenesis syndrome and, possibly, with the unexplained male infertility that accounts for the 30–40% of men with abnormal semen parameters [47]. In an animal model of hypogonadal mice, androgen administration not only initiated TJ formation, it also stimulated an increase in testicular weight and of the diameter of seminiferous tubules and initiated in some tubules the production of post-meiotic elongated spermatids [48]. Sperm cells were not observed.

All three peptides, such as testosterone, had a positive effect on the formation of TJs between neighboring 93RS2 Sertoli cells, as demonstrated by their stimulatory effects on TER. Abrogation of ZIP9 expression by siRNA prevented all effects associated with TJ formation, indicating that this transporter is an essential androgen receptor of male gonads whose expression is directly regulated by testosterone [49,50]. Androgen/ZIP9 interactions drive senescence in regressed vole testes [50], possibly also by being directly involved in the BTB dynamics that maintain and safeguard male fertility. Taking into consideration the results presented here and the fact that testosterone actions via the classical AR are essential for spermatogenesis, the specific targeting of the ZIP9 androgen binding site with the tetrapeptides used in the current study in combination with testosterone treatment might prove to be useful in the treatment of BTB-related male infertility. It remains to be seen whether the negative feedback mechanism can be circumvented. Experimental work involving the hypogonadal mouse model may help to clarify this possibility.

## 5. Conclusions

All synthetic peptides interact specifically with the androgen binding site of ZIP9 localized at the extracellular side of this plasma membrane-embedded protein. They mediate signaling cascades in Sertoli cells that are identical to the so-called non-classical signaling pathway of testosterone. The induction of this signaling cascade by the peptides stimulates the expression of TJ-associated proteins such as ZO-1 and claudins and the formation of TJ. All of these effects depend on the presence of ZIP9, and a different androgen receptor is not involved. The results presented here indicate that the peptides bear the potential to serve as testosterone surrogates to treat BTB-associated male infertility; their use may avoid the negative effects of conventional testosterone replacement therapy such as polycythemia, cardiac hypertrophy or infarction, or even worsened male infertility.

## Data Availability

From the corresponding author, upon reasonable request.

## Supplementary Materials

The following supporting information can be downloaded at: https://www.mdpi.com/article/10.3390/biology11010055/s1, Figure S1: Western blots used in Figure 3 of the manuscript. Figure S2: Western blots used in Figure 5 of the manuscript. Table S1: Antibodies used in the investigation, their providers, and the concentrations used for WB or IF.
