# Supplementary material for: Tetrapeptides Modelled to the Androgen Binding Site of ZIP9 Stimulate Expression of Tight Junction Proteins and Tight Junction Formation in Sertoli Cells
Source: Biology (Basel). 2021 Dec 31;11(1):55. doi: 10.3390/biology11010055 (PMC8773409; doi:10.3390/biology11010055)
Supplement: Supplementary file 1 [file biology-11-00055-s001.zip › biology-1503136-SUPPLEM-final.pdf]

Supplementary Table 1: Antibodies used in the investigation, their providers, and the concentrations used for WB or IF.

Antibody	Provider	Cat-No.	Suitable Application acc. to provider	Antibody concentration (ABC) or dilution used
Myogenin	Invitrogen	MA5-11486	WB, IHC, IP	ABC: 0.5 mg/mL<br>Used: 3μg/ml
Beta Actin	Cell Signaling	4970S	WB, IHC, IF, FC	WB 1:1000
P-FAK	Cell Signaling	3284S	WB, IP	WB 1:1000<br>IF 1:250
Alkaline Phosphatase	Santa Cruz	Sc-271431	WB, IHC, IP, IF	IF 1:250
P-Erk1/2	Cell Signaling	4370S	WB, IHC, IP, IF	WB 1:2000<br>IF 1:200
T-Erk1/2	Cell Signaling	9102S	WB, IHC, IP, IF	WB 1:1000<br>IF 1:250
P-CREB	Cell Signaling	9198S	WB, IHC, IF, FC	WB 1:1000<br>IF 1:800
Claudin-1	Novous	NBP1-77036	WB, ICC, IHC, IF	ABC: 1 mg/mL<br>Used: 20μg/ml
Claudin-5	Santa Cruz	Sc-374221	WB, IF, IP	IF 1:200
ZO-1	Cell Signaling	5406S	WB, IF	WB 1:1000<br>IF 1:250

WB = Western blot; FC = flow cytometry; ICC = immunocytochemistry; IHC = immunohistochemistry; IF = immunofluorescence; IP = immunoprecipitation.

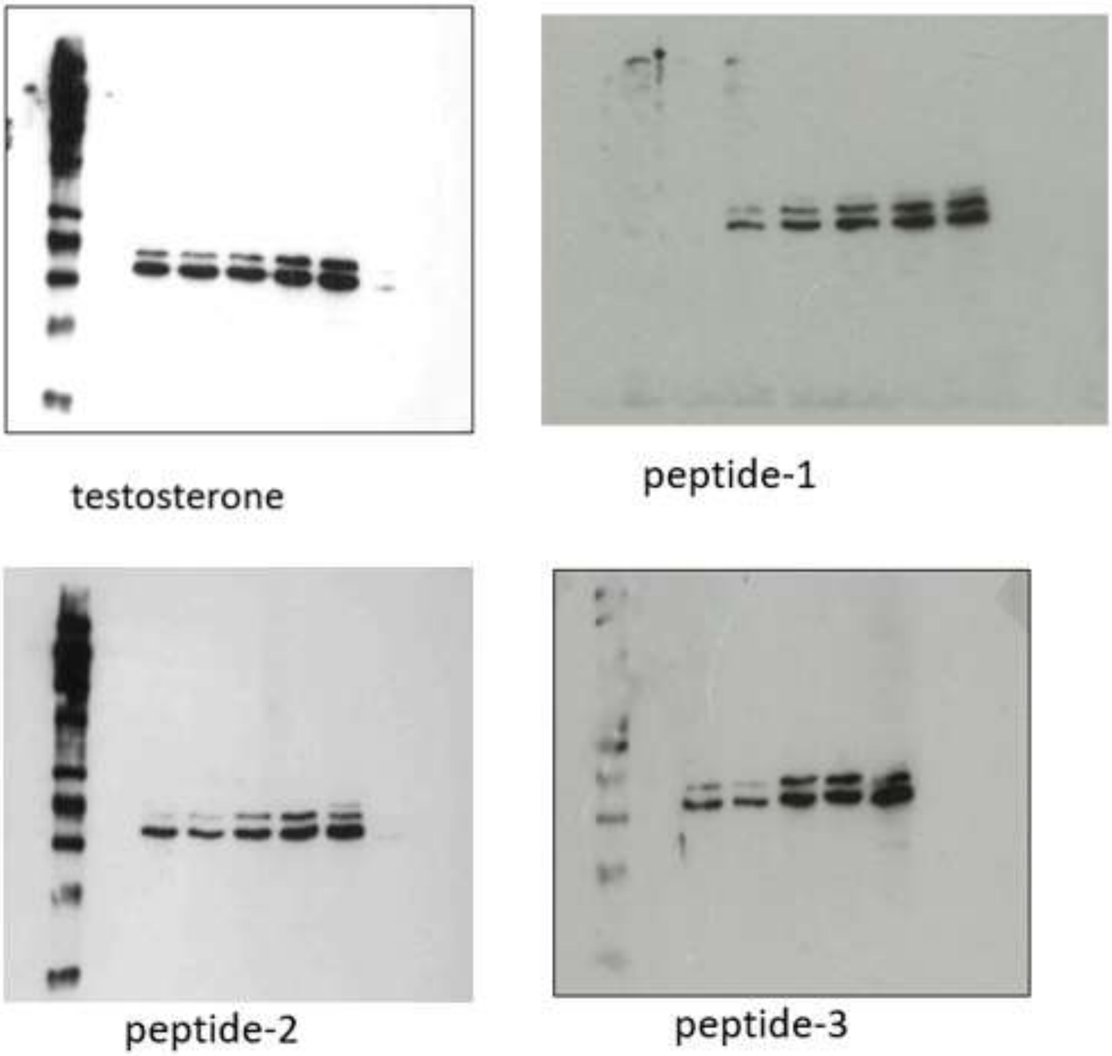

Figure S1: Western blots used in Figure 3 of the manuscript.

Activation of Erk1/2 by Testosterone, peptide-1, peptide-2 and peptide-3.

Concentrations for T are 0, 0.1, 1, 10, 100 nM.

Concentrations for each of the peptides are: 0, 0.1, 1, 10, 100 μM.

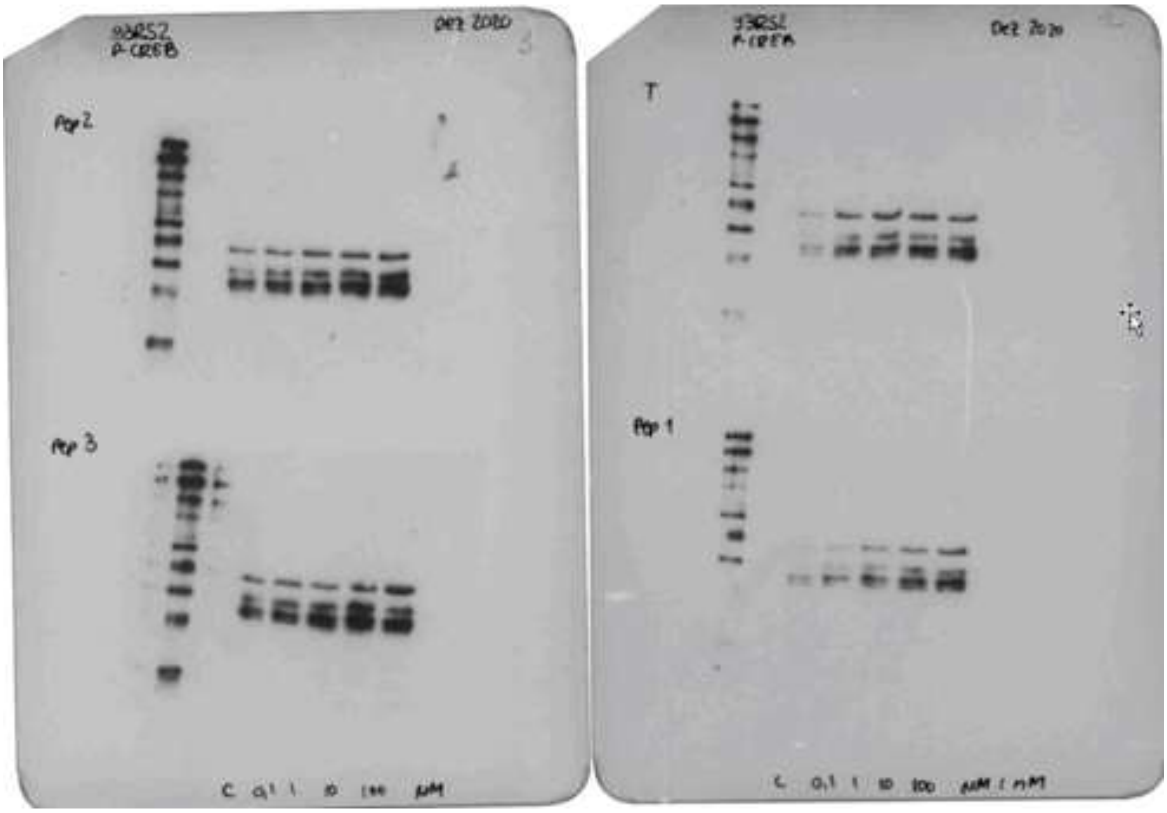

Figure S2: Western blots used in Figure 5 of the manuscript.
Activation of CREB/ATF-1 by Testosterone, peptide-1, peptide-2 and peptide-3.
Concentrations for T are 0, 0.1, 1, 10, 100 nM.
Concentrations for each of the peptides are: 0, 0.1, 1, 10, 100 μM.
